# Trends and Progress on Antibiotic-Resistant *Mycobacterium tuberculosis* and Genes in relation to Human Immunodeficiency Virus

**DOI:** 10.1155/2023/6659212

**Published:** 2023-11-30

**Authors:** N. G. Mbewana Ntshanka, T. A. M. Msagati

**Affiliations:** College of Science, Engineering and Technology, Institute for Nanotechnology and Water Sustainability University of South Africa Science Campus Roodepoort, 1709 Johannesburg, South Africa

## Abstract

Human immunodeficiency virus/acquired immunodeficiency syndrome (HIV/AIDS) and tuberculosis (TB) are among the infectious diseases that cause high rates of mortality worldwide. The epidemiology of antibiotic resistance in correlation to people that live with TB and HIV has not been thoroughly investigated particularly in South Africa. Numerous cases of multidrug-resistant TB (MDR-TB) and extensively drug-resistant TB (XDR-TB) have been announced immensely worldwide. The spread and control of the MDR-TB pandemic due to unsuccessful treatment is one of the most serious public issues of concern, and this challenge is of international interest. Despite all measures that have been executed to overcome the challenge of MDR-TB in recent decades, the global MDR-TB trends have kept on accelerating with more and more people becoming victims. This is attributed to the abuse, misuse, and overuse of different antibacterial agents in human medicine, animal farms, and agricultural activities which serve as a wellspring for the evolution of antimicrobial resistance within the population. Over and above, the impetuous evolution, mutation, and the transfer of resistant genes via horizontal gene transfer are well-known contributive factors towards the antimicrobial resistance problem. Among the public health concerns in the world currently is the ever-increasing problem of antibiotic resistance which outpaces the progress of newly developed antimicrobials. The propagation of antimicrobial resistance (AMR) is even more amplified in areas where the pressure of antimicrobial resistant pathogens is elevated, and hence the population with ubiquitous HIV and AIDS is considered the hotspot. This review therefore aims to give in-depth coverage on the trends and the progress on the development of TB and HIV-resistant strains, highlight strategies to solve the problem, and accentuate the repercussions of the COVID-19 epidemic on the AMR.

## 1. Introduction

Two decades ago, several outbreaks of infectious diseases have severely impacted the health status of many communities, such that in 2019, the World Health Organization (WHO) announced the top 10 diseases that threatened the global health [1]. The diseases in question included influenza, human immunodeficiency virus (HIV), dengue, Ebola [2], acute respiratory syndrome (SARS-CoV) (with outbreaks in 2002-2003), Ebola (with outbreaks in 2014–2016), H1N1 flu (swine flu) (with the outbreaks in 2009-2010), Zika virus (2015-2016), and now SARS-CoV-2 (erupted in late 2019) [1, 3]. HIV being on the top 10 diseases together with its synergistic associate, tuberculosis (TB), is in the midst of lethal communicable diseases worldwide that lay claim to about 1.6 million and 1.3 million lives year after year, respectively [4]. To date, TB persists to be amidst the causes of death worldwide, with approximately ten million five hundred thousand tuberculosis patients and approximately one million six hundred thousand deaths in 2018 [5]. In 2020, 10.1 million persons were assessed to be infected with *Mycobacterium tuberculosis*; of those, about one million five hundred succumbed to the disease and this was attributed to the impact of COVID-19. In 2021 only, approximately 1.6 million people died, the number is inclusive of 1.4 million deaths of people that were HIV-negative and the 187 000 deaths from HIV-positive people. However in 2020, the mortality count increased to 1.5 million from 1.4 million that was recorded in 2019. The global estimates of TB deaths between 2019 and 2020 reversed the gradual decrease that was achieved between the years 2005 and 2019 [6]. The reverse owes to the impact of COVID-19, and the numbers are anticipated to continue inclining globally even in 2023 [7].

South Africa (SA) has been listed among the countries with ubiquitous debilitating sicknesses in the world. Sudre et al. estimated that TB has spread among 33.33% of the world's populace [8] and more than eight million infections take place annually [9], and this makes it very challenging to manage. Currently, there are now 2 more tremendous alarms to the universal tuberculosis control, and they include the HIV pandemic and the increasing prevalence of multidrug-resistant tuberculosis. The HIV-1 infection threatens the progress of the tuberculosis control plans especially in countries that are mostly affected by AIDS, notably sub-Saharan Africa [10].

The treatment of tuberculosis and HIV requires the administration of antibiotics. However, the present global epidemic of antimicrobial resistance (AMR) is posing a serious health hazard. The advent of HIV drug resistance is a great hindrance to the successful treatment of antiretroviral (ARV). Under normal circumstances, ARV therapy curbs the HIV-1 viremia; however, HIV-1 may revive due to the widespread nature of AMR [11]. Moreover, the utmost severe condition of multidrug-resistant tuberculosis, known as extensively drug-resistant tuberculosis (XDR-TB), has been recognized and described as an adverse global health challenge, and it is therefore extremely important to investigate further [12]. The WHO has recently announced the risk of depleting all the therapeutic regimens due to the increased cases of antibiotic resistance (ARB) [13].

This raises a serious health concern especially taking into consideration the high-rise incidences of debilitating diseases like TB and HIV. A probable synergic syndicate linking AIDS, TB, and VRE infections brings uncertainty and dread [14]. Consequently, a novel environment which presents a distinct selective pressure that leads to rigorous probabilities of the emergence of ARB is presented in populations with high abundance of active AIDS [15]. As a result, the AMR develops and multiplies quickly [16] in population with prevalent HIV/AIDS (>25% prevalence) [17] because of the constant consumption of antibiotics which promotes the development and propagation of AMR [18]. The repercussion is aggravated by the limitation of the access to the antimicrobials as well as the noncompliance to therapeutic regimens [19]. Given the circumstances, the strains that are partially resistant exploit their survival probabilities and evolve to prominent resistant pathogens [20]. Since AIDS-prevalent regions are considerable reservoirs of AMR, the emergence of resistance is not substantial to that population but generates a global health concern [21, 22], thus prompting the need for intervention strategies.

## 2. Methods Employed in the Collection of Data and Information

This review is inclusive of all the related scientific publications and reports that were collected by searching on the electronic databases using the following keywords: antimicrobial resistant bacteria (ARB), *Mycobacterium*-TB, HIV, MDR-TB, XDR-TB, antibiotic resistance *Mycobacterium*-TB, *Mycobacterium*-TB in wastewater treatment plants, and public health. The search was conducted using Google Scholar, PubMed, Scopus, NCBI, and ScienceDirect for the applicable studies starting from the year 2000 to 2022. The search was based on national surveys and pinpointing a few countries according to their data submitted to WHO.

## 3. Results or Findings

The analysis of this paper included surveillance and findings reported within at least one decade by the World Health Organization and public health.

### 3.1. The Epidemiology of TB and HIV Worldwide

#### 3.1.1. The Emergence of Drug Resistance

**Figure 1:**
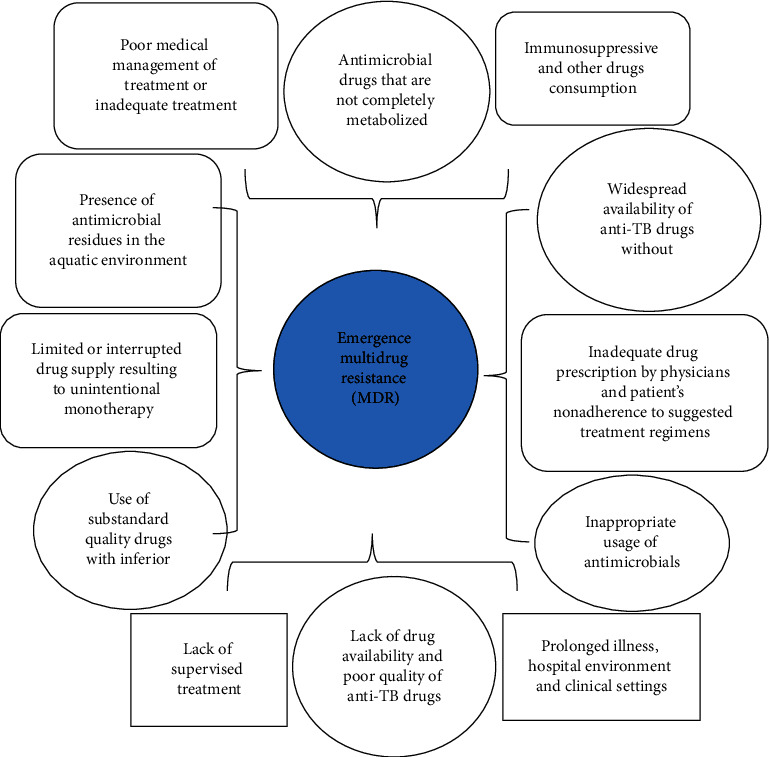
Diagram indicating factors that effectuate the evolution of resistance and thereby result in multidrug resistance [1, 23, 24].

Although microbial evolution happens naturally, some factors such as incorrect regimen of drug consumption elevate pressure and thereby promote the rate and magnitude of resistance [1, 23, 24]. The expeditious development of drug resistance is also associated with antimicrobials that are prescribed inadequately and patient's poor medical adherence to the recommended prescription regimens which result in subsequent transmission of AMR strains within the population. More variables like poor medical management of treatment, shortfall in establishing the suggested therapies, shortfall of therapeutic management, and insufficient medication resulting in unintentional monotherapy add even more pressure. All these promoting factors for the emergence of MDR are displayed in Figure 1.

In the past, when many outbreaks of MDR were reported in different regions, it was not considered as one of the major problems of the world until the 1990s [25–27]. It has now been reported to spread across the countries [28]. Contemporaneously, the rapid increase of MDR is perceived as fabricated issue [28] because it can be managed with good measures and policies put in place for their adherence. The increasing rate of resistance has caused AMR to be extolled to be among the greatest twenty-first century's problems [29]. The apprehension of the AMR increase has resulted in the formation of the Global Antimicrobial Resistance Surveillance System (GLASS) in 2015 by the WHO. The aim of GLASS is to share knowledge globally to reinforce facts and assist in reaching agreements nationwide as well as internationally [1, 30].

#### 3.1.2. Drug Resistance in Tuberculosis Cases

Tuberculosis is an illness caused by *Mycobacterium* strain which is detected among millions of human beings yearly and categorized alongside the HIV posing a huge threat globally as it is among the top sicknesses that result in death worldwide [31]. Multidrug-resistant TB is described as the resistance that originates from a strain that poses resistance to isoniazid and rifampicin, while extensive drug resistance is described as the resistance that originates from MDR-TB strain that poses resistance to any fluoroquinolone and at least one of the three second-line injectable antimicrobials (SLIDs) [32–34]. The emergence of XDR-TB is anticipated to be arbitrated solely by chromosomal mutations, which influence either the bacterial enzymes that activate pro-drugs or the drug target itself [35]; however, Nimmo et al. suggested otherwise [36].

In the last decade (2010), approximately 8.8 (8.5–92) million TB cases were reported and about 1.1 million patients passed away due to TB infections; however, 0.35 million patients were TB/HIV-positive, and the rest were HIV-negative patients [37]. In Asia-Pacific region, HIV/TB infection is not considered as a significant cause of the TB pandemic despite the high rate of infection to specific groupings [38]. They highlight that in 2013, 6.3% of the patients tested had been infected with tuberculosis and this called for a direct investigation because HIV/AIDS test has been standardized for patients with tuberculosis infection. During meta-analysis that was conducted on pupils with multidrug-resistant TB, the results revealed that the success of the treatment was still unsatisfactory as only 62% of patients were successfully treated, 17% reverted, 9% succumbed to the infection, and in 7% of patients, the infection bounced back. The results for the extensively drug-resistant TB were also inadequate as they accomplished only 40% of full recovery, 22% either did not complete the treatment or had setbacks, 16% backed out, and 15% succumbed to the sickness [38]. The World Health Assembly endorsed the “Global Strategy and Targets for Tuberculosis Prevention, Care, and Control” in 2014, which aims to eradicate the challenge of high infections of tuberculosis by 2035 [39, 40].

In 2015, World TB Day raised public alert that till this day tuberculosis continues to be a “global emergency.” It is therefore essential that every country put control activities in place outlining its own epidemiological situation. Despite all the efforts that have been made by WHO, multidrug-resistant TB has accomplished only 48% treatment success rate in the globe, and this is attributed to the feeble systems of the healthcare centers. The Global Tuberculosis Report issued in 2014 suggested that about 3.5% of the cases that were new and 20.5% of the patients that were formerly treated in 2013 had multidrug-resistant strains [41, 42].

In 2016, only 7 countries were responsible for the entire 64% of TB cases. The countries included India with the highest percentage, succeeded by China, then Philippines, followed by Nigeria and Pakistan, and then SA. From 64%, a sum of 1.7 million patients succumbed to the tuberculosis disease, and of those, 0.4 million were HIV-positive [43, 44]. On average, the percentage of multidrug-resistant TB infections and extensively drug-resistant TB in 2016 was approximately 6.2%, and this percentage was lesser than the percentage of the previous years which was 9 and 9.7%; however, the cases of the extensively drug-resistant TB were still escalating [44].

In 2018, WHO reported that not all drug-resistant tuberculosis patients were identified, and moreover, from those, only 51% were examined for the rifampicin resistance. Furthermore, among the estimated half a million patients that were accounted for MDR/RR-TB, only 33% received treatment. Therefore, drug-resistant tuberculosis remains a health priority [45] and the infection is estimated to be transmitted to at least nineteen million pupils [45, 46].

#### 3.1.3. Drug Resistance in HIV/AIDS Cases

Tuberculosis, a global health problem, is aggravated by the coinfection of HIV which claimed about 73% of South African active TB cases in 2013 [47, 48]. HIV infections have been shown to advance the underlying TB infection to tuberculosis disease. HIV-positive patients stand 21–34 chances to acquire tuberculosis infection in comparison to people that are HIV-negative [49–51]. Plenty of antimicrobial drugs such as ARV and anti-TB antibiotics are consumed to manage pandemics such as TB and HIV. However, the abundance of the drugs has become a major problem because they can be detected even in aquatic environment leading to an emergence of resistance [52]. Consequently, the success of the ARV and anti-TB therapy is strongly obstructed by the existence of TB/HIV drug resistance. At first, HIV-1 viremia is curbed by antiretroviral therapy; however, due to AMR strain, the sickness bounces back to many patients [53]. It is therefore important to monitor drug resistance as it plays an essential role in supervising people that are on antiretroviral therapy as it will regulate the upcoming medical regimes.

#### 3.1.4. Mechanisms of Drug Resistance

**Figure 2:**
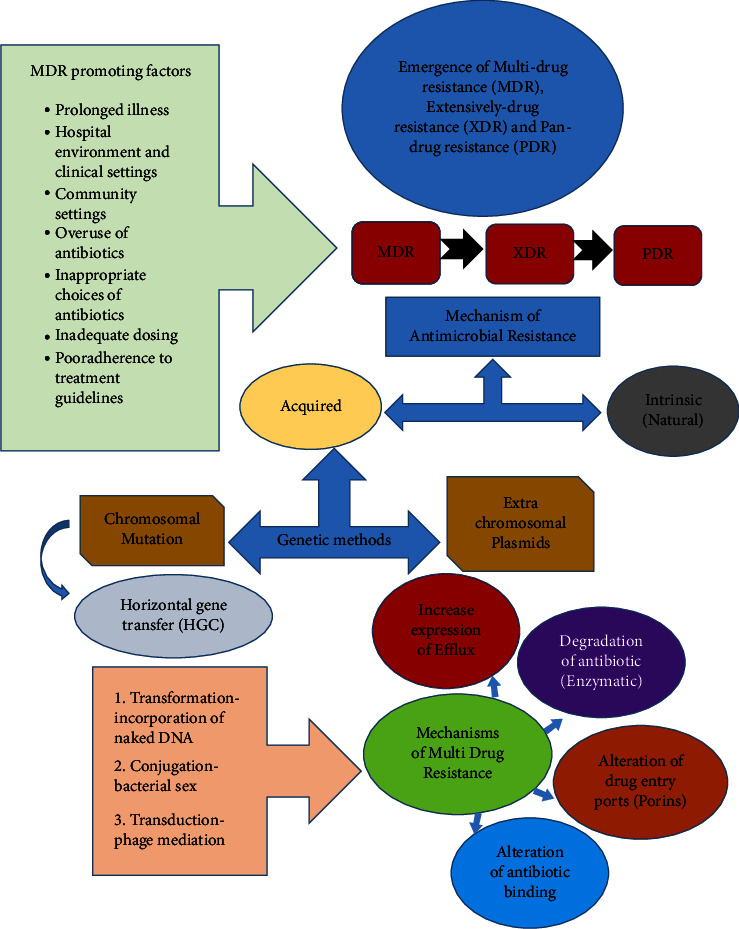
Mechanistic basis of antimicrobial resistance [54, 55].

Resilient bacteria have developed strategies to resist the presence of antimicrobial compounds; hence, they are often regarded to be “intrinsically” resistant. Resistant microbes use two mechanisms to withstand and resist the presence of antimicrobial agents which are mutations and attaining foreign DNA coding using horizontal gene transfer (HGT) mechanism. Nonetheless, intrinsic bacterial resistance quandary is not the main problem in the discussion of AMR. Relatively, “acquired resistance” of the bacterial community that was at first inhibited by antibiotic compounds is the major obstacle in most therapies. The acquired resistance is developed by mutation in genetic code or due to the accretion of resistant genes in the surrounding that are probably inherited from intrinsic immunity present in the environment [54]. The masterplan that bacteria use to acquire external genetic material is via (i) transformation mechanism which includes incorporation of naked DNA, (ii) transduction mechanism which includes phage mediated, and (iii) conjugation which involves bacterial “sex.” In HGT, transformation is considered the simplest method; however, it is very difficult to manage clinically as the bacteria are capable of absorbing DNA “naturally” and thereby acquiring resistance. Conjugation methods use mobile genetic elements (MGEs) to channel and distribute the important as vehicles to share valuable hereditary details even though unmediated transmission between chromosomes is always identified [55]. After resistant mutants emerge, a segment of one-celled organism that is obtained from a susceptive community evolves through mutation and thereafter modifies the drug and eventually withstands the presence of antimicrobial molecules. The antimicrobial-resistant cells alter the antibiotic molecules using these mechanisms: (i) altering the drug target by reducing the drug's affinity, (ii) reducing the drug intake, (iii) stimulation of efflux mechanisms to eject the toxic fragments, or (iv) universal shifts.  Figure 2 shows the mechanisms associated with XDR-TB.

#### 3.1.5. Mechanisms of Drug Resistance in *Mycobacterium tuberculosis*

Contrary to other bacterial microorganisms which obtain AMR via HGT and thereby are capable of deactivating antimicrobials through extrachromosomal resistance genes, *M. tuberculosis* uses three mechanisms, namely, activator mutations, target-based mutations, and efflux pumps [36]. In essence, the *M*. *tuberculosis* mechanisms reduce the permeability of the antimicrobials, use the *M*. *tuberculosis* enzymes to modify the antimicrobials, extrude all the antimicrobials that are capable of crossing the *M*. tuberculosis envelope by efflux pump, and alter its gene expression for the adaptation of the antimicrobial's reaction. Moreover, the envelope cell of *M*. *tuberculosis* is made up of four layers which furnish a support structure and shield to osmotic variations. The four layers of the envelope are inner plasma membrane, periplasmic space, a core enfold that comprises peptidoglycan (PG) covalently linked to arabinogalactan (AG) and mycolic acids (MAs), the peripheral lipid layer created by noncovalently joined lipids and glycolipids that includes trehalose dimycolate (TDM), phthiocerol dimycocerosates (PDIMs), mannose-capped lipoarabinomannan (ManLAM), sulfolipids (SLs), phosphatidyl-myo-inositol mannosides (PIMs), and phenolic glycolipids (PGLs), and the outermost layer which is normally referred to as capsule. The *M*. *tuberculosis* envelope not only supports the structure but also takes part in the immunomodulation of the bacterium-host crosstalk, where supposedly, many cell envelope outer molecules are known to take part in phases of the infection with major impact in *Mycobacterium* immunological and evolution of AMR [56].  Table 1 presents the resistant mutation genes and the antitubercular drugs of their correspondence. On the list, katG and inhA are responsible for about 64.2% and 19.2% of isoniazid resistance, respectively, while inhA combined with ahpC-oxyR is responsible for 84% of isoniazid resistance globally [58].

### 3.2. Status of Drug Resistance in TB and HIV

#### 3.2.1. Trends and Patterns of TB and HIV/AIDS Antimicrobial-Resistant Pathogens

In the year 2000, the WHO estimated that fifty million pupils in the globe were infected with multidrug-resistant TB, and about 273 000 cases of 8.5 million new tuberculosis infections were caused by MDR-TB [28]. In 2002, the WHO thereby declared that the resistance of antimicrobials has become a crucial issue in therapies for infectious sicknesses and diseases like HIV, TB, pneumonia [59], malaria, gonorrhea, and diarrhea. WHO further stipulated that up to 75% of antimicrobials are issued incorrectly and nonadherence is observed even in academic healthcare centers of the underdeveloped countries [7]. This therefore accelerated the drug resistance rapidly and set off a threat to diseases that currently have no cure such as HIV/AIDS. It is estimated that nearly a hundred million regimens of antimicrobials are authorized by physicians in their practicing rooms yearly based on the report from CDC. The CDC further elucidated and claimed that approximately half of the antimicrobials are prescribed for patients with cold symptoms, cough, and other viral infections [7]. In SA, the WHO estimated about 1.8% of MDR-TB infections, while the estimation increased to 6.7% with cases that were previously treated [60] in 2002 worldwide survey [61]. The Joint United Nations Program on HIV and AIDS [62] estimated that about 39.4 million people are infected with HIV and over three million people have succumbed to this sickness. UNAIDS further exclaims that the disease is a pressing international health problem that calls for attention [63].

About nine million four hundred thousand tuberculosis infections were recorded globally by WHO in 2008 and one million and eight hundred thousand victims died from TB disease, and this equates to 4.500 per day and Asia was reported to account for the largest value of multidrug-resistant TB incidents [64]. In that same year, 2008, WHO raised a concern with regard to the universal intensifying increase of drug-resistant tuberculosis pandemic [65]. They estimated about 440,000 incidents of multidrug-resistant TB that were resistant to the first-line agents (isoniazid and rifampicin) in the year 2008. From these cases, about 40, 000 cases were detected to be extensively drug-resistant TB and multidrug-resistant TB, and more resistance is associated with fluoroquinolone as well as the injectables such as capreomycin, kanamycin, or amikacin [60, 62, 66].

**Figure 3:**
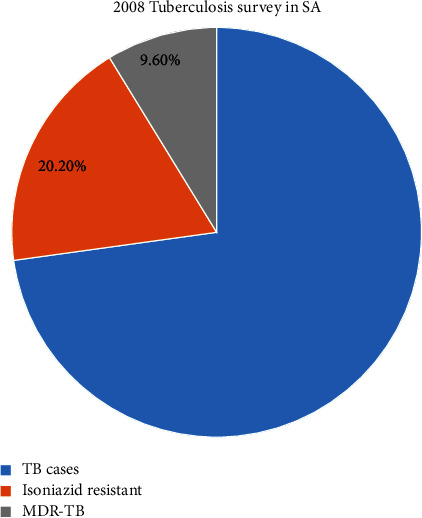
*Mycobacterium tuberculosis* infection national survey in 2008 [64].

In January 2010, more extensively drug-resistant TB cases were recorded among 58 countries globally [62, 64]. SA was also classified to be among the countries that had the highest weight of multidrug-resistant TB with approximately 13,000 cases in 2008 [64]. Of these cases, the statistics revealed that only 8200 cases were recorded with multidrug-resistant TB or extensively drug-resistant TB by the NHLS insinuating a diagnostic percentage of 63% [61]. Furthermore, only up to 50% of the detected cases were enrolled on multidrug resistance therapy plan in 2009 [67].  Figure 3 illustrates the 2008 nationwide survey that was conducted in SA in 2008 which revealed that 20.2% of the informed patients with tuberculosis were resistant to isoniazid and almost half of these patients (9.6%) had multidrug-resistant TB [64].

**Figure 4:**
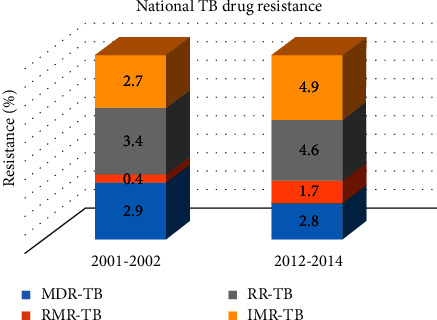
National tuberculosis drug resistance surveillance between 2001-2002 and 2012–2014 [69, 70].

This was an indication of a great escalation (3-fold) since the year 2002, when only 3.1% of all tuberculosis infections detected had multidrug-resistant TB [60, 67, 68]. The National Health Laboratory Service (NHLS) supported these findings which revealed a gradual escalation of patients with multidrug-resistant TB from 2004 [60]. In contrast to the multidrug-resistant TB ubiquity, the pace of any rifampicin-resistant TB (RR-TB) ubiquity has considerably escalated in 2014 survey with an overall prevalence of 4.6% compared with 3.4% that was in 2002 survey. The survey of rifampicin mono-resistance (RMR) also raised some concerns when compared to the previous survey which revealed a minimal ubiquity. In the previous survey, RMR was below 0.5% and yet has increased up to 1.7% in the 2014 survey. It was also noted that isoniazid mono-resistance (IMR) has also increased from 2.7% to 4.9% since the previous survey (South African Tuberculosis Drug Resistance Survey 2002–2014). NHLS data predicted that 6.3% of the recorded multidrug-resistant TB incidents are extensively drug resistant, while the predictions from WHO are also gloomy with approximately 10.5% of extensively drug-resistant TB among the multidrug-resistant TB patients [62, 64].  Figure 4 illustrates the TB drug resistance 2002 survey versus 2014 survey.

Gerona et al. [71] reported that multidrug-resistant TB and extensively drug-resistant TB threaten the public health even though the incidence of global TB is decreasing approximately by 2% per year. About 0.5 million new infections of AMR are creating a high rate of morbidity, mortality, and health system interference compared to other diseases that are inhibited by antimicrobials [71, 72].

About 450 000 new patients of multidrug-resistant TB were recorded in 2012 and this resulted in 170 000 deaths [73]. MDR-TB accounted for 5.7% of TB cases, and this was mainly resistant towards isoniazid and rifampicin worldwide as predicted by WHO [74]. In the same year, 2012, a range of 8.3 to 9.0 million TB cases (approximately 8.6 million) which were equivalent to 122 cases per 100, 000 populations were estimated worldwide [75]. WHO [76] claimed that the total number of cases has been decreasing though at a slow rate since early 2000. Many of these cases were in the Western Pacific (58%) and the African region (27%); fractions of incidents were recorded in the Eastern Mediterranean region (8%), European region (4%), and the region of the Americas (3%) [77]. India demonstrated a high number of TB cases ranging from two million to 2.4 million correlating to 26% incidents of globe followed by China with a range of 0.9 million–1.1 million and then SA ranging from 0.4 million to 0.6 million. The rate of incidence varied from one country to another with high-income countries such as those in Western Europe, Canada, the United States of America, Australia, and New Zealand predominating lowest rates. It is estimated that in SA and Swaziland, at least 1% of the human population gets infected with tuberculosis every year [78].

One million incidents of tuberculosis were recorded from people that are HIV-positive globally in 2013. About 78% of these cases were in Africa. In 50% of HIV-positive patients, their first-line manifestation is TB and death rate is relatively high in sub-Saharan Africa while in good few countries, this rate is recorded to be immoderate by 50% [79, 80].

Consequently, by the year 2014, WHO had reported nine million new tuberculosis incidents and 1.5 million tuberculosis deaths [78, 81]. The World Health Organization further predicted that >25% of *Streptococcus pneumoniae* in all its six regions are resistant to penicillin and >50% of *Escherichia coli* are resistant to third-generation cephalosporins in five out of all its six regions as demonstrated in Table 2. The resistance of beta-lactam antibiotics in Africa is reported to be up to 100% [83]. WHO also declared that some organisms are resistant to vancomycin, third-generation cephalosporins, clindamycin, and carbapenems which were the last resort. This led WHO to recommend for innovation of extra anti-drug in 2017 [4]. CDC responded to this global threat by launching the Antimicrobial Resistance Surveillance Network (AMRSNET) which is currently in collaboration with the World Health Organization to support AMR surveillance in Africa [84].

2014 World Health Organization's global report about antimicrobial resistance revealed great voids in the inspection that was conducted in different countries that include Zimbabwe for antimicrobial resistance pathogens which are responsible for malaria, HIV, and TB. A creditable technologically recently developed laboratory accredited by the South African National Accreditation System (SANAS) in Zimbabwe reported on the trend of antibiotic resistance that is accelerating by an average of 0.7% ranging between 73.9% and 74.6% towards amoxicillin in 2011 to 2015 [82]. During the 2014 MDR-TB and XDR-TB worldwide screening, WHO established that 480,000 incidents fall under multidrug-resistant TB, and a number of these incidents are found in India, China, and the Russian Federation with China being the leading country [85–87]. In 2014, SA recorded about 35, 000 MDR-TB cases [88, 89].

In 2015, approximately 580,000 infections advanced to multidrug-resistant TB globally, and among them, hundreds were XDR-TB. Incidents of totally drug-resistant TB (TDR-TB) were also recorded [32, 90, 91]. WHO suggested that SA is included in the countries that are recording high incidents of multidrug-resistant TB in the globe and this is associated with the perceptible increase of multidrug-resistant TB cases that are recorded [89]. WHO estimated about 37 000 TB cases with a mean incidence of 107 cases per 100 000 inhabitants in the same year (2015) [92].

WHO estimated about 10.4 million new incidents of tuberculosis in 2016 and further recorded 1.3 million deaths that resulted from TB infections as well as 0.37 million deaths from tuberculosis infections among the HIV-positive patients [93–96]. In the same year, 2016, WHO also reported about 36.7 million people that are HIV-positive, 1 million patients who passed away due to HIV-related sicknesses, and 1.8 million patients that are newly infected worldwide. WHO further explained that the most affected area is the African region which is responsible for about 66% (25.6 million people) of new HIV infections globally [97]. SA has been reported to be experiencing the burden of RR-TB as well as MDR-TB [98]. Among all cases that were reported globally in 2016, approximately 490,000 cases were diagnosed to be multidrug-resistant TB and about 110,000 personnel showed resistance towards rifampicin [99]. Furthermore, 6.2% cases of the 110,000 were predicted to be XDR-TB cases instead of MDR-TB cases [89, 100]. According to the 2017 report by the Tuberculosis Facts Organization, South Africa recorded 19,000 cases of MDB-TB [89].

WHO reported an estimation of 10 million new tuberculosis infections and 1.6 million deaths that are associated with the sickness [100, 101]. Of these cases, SA was reported to be responsible for 322,000 cases of active TB [102], but the cases reported had drastically decreased from 400,000 to 300,000 in 2014 [89, 103]. WHO stipulated that regardless of the decrease in TB infection in the globe and death rates in comparison to the previous years, more efforts are required to achieve the 2030 set targets of Ending the Tuberculosis Strategy: which is meant to reduce the tuberculosis death rate at least by 90% and tuberculosis infection rate by a minimum of 80% [102, 104]. In 2018, WHO declared about ten million cases with frightening recordings of RR among 0.5 million new incidents upon which 78% were identified as multidrug-resistant TB1. An estimation of 3.4% of all new cases and 18% of all previously treated cases was reported globally [105]. WHO further deduced that in underdeveloped countries, TB is prone to result in deaths compared to other communicable diseases [106, 107] and further elucidated that 53,620 TB cases were pointed out in the Middle Eastern and North African (MENA) region which includes the countries of Iraq, Egypt, and Sudan [108, 109]. The rapid increase of ARB has led WHO to designate global accessibility to DST as rapid test for rifampicin at minimum as well as for fluoroquinolones among all the rifampicin-resistant TB cases as an initial test to determine whether the patient is resistant to antimicrobials prior to TB therapy regime, and this was recommended to all patients [110]. These drugs were selected because isoniazid and rifampicin have major influential antimicrobials and foundation of antituberculosis therapy. It is worth noting that the therapy of multidrug-resistant TB case is way more problematic and 100% pricier than the case with no AMR [15].

**Figure 5:**
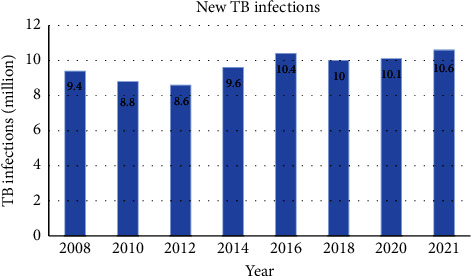
Global annual tuberculosis infections depicting the surveillance spanning for over a decade [7, 77, 94, 100, 117–120].

6.2 million South African personnel are estimated to be HIV-positive [111], and as per the National Department of Health (NDOH) report, 50% of these pupils are signed up for ARV therapy [112]. WHO elucidated that patients that are coinfected with both HIV and TB are more prone to succumb to the tuberculosis disease and not only that but very sensitive to other infectious diseases which includes *Pneumocystis jirovecii* pneumonia because certain anti-drugs are dispensed to all HIV-positive patients including the ones that have CD4 count of 500 cells/ml, also to infants and as well as adults as post-exposure prophylaxis to HIV infection [113]. This causes South Africa to be among the countries with highest administration of ARVs, anti-TB, and other anti-drug per unit of population while containing the HIV/AIDS and TB scourge [114, 115]. To date, WHO has published “*Global Tuberculosis Report 2019*” that clearly speculates that most of the regions with heavy tuberculosis burden were already far off to attain 2020 milestones of End TB strategy [116].  Figure 5 illustrates the trends of tuberculosis infections between the years 2008 and 2021 which cover at least one decade of surveillance.

In 2019, the patients who were enrolled for multidrug-resistant/rifampicin-resistant TB course globally were 181 533. This number went down to 150 469 in 2020; however, it slightly increased by 7.5% in 2021 and this took the numbers to 161 746. It was noted that most of the enrolled patients were adults. The rate of achievement for multidrug-resistant/rifampicin-resistant TB treatment using second-line regimens in 2019 was 60%, a great improvement of 50% that was obtained in 2012. In most countries, a number of patients that were identified to have DR tuberculosis were monitored for hostile events throughout the year 2021. In the year 2021, 10 countries were responsible for 72% of the gap between the numerical values of patients who registered for the MDR/RR-TB treatment and the numerical values of patients who were identified to have multidrug-resistant/rifampicin-resistant TB globally. In the same year, 2021, most countries resorted for bedaquiline as part of the multidrug-resistant and extensively drug-resistant TB treatment. This resulted in about 109 countries that were taking all-oral longer regimens which increased from 92 in 2020 and 86 in 2019. The countries that were using shorter treatment regimens for MDR/RR-TB also increased in 2021 from 65 countries in 2020 to 92 countries [121]. Overall, an estimation of 10.1 million personnel was diagnosed with tuberculosis in 2020 and this number increased by 4.5% resulting to 10.6 million cases in 2021 globally [7].

#### 3.2.2. Global Action Plans by the World Health Organization (WHO)

In 2013 annual global tuberculosis report, the WHO estimated about nine million people who were infected with tuberculosis; from that, 1.2 million (14%) were estimated to be people that are HIV-positive. WHO further explained that an unpropitious rise in new cases of MDR-TB and XDR-TB was observed globally, and the recent incidents of MDR-TB were estimated to be 480,000 in 2013 [122]. World TB Day 2015 raised public alert that tuberculosis is still a “global emergency” and claiming 1.5 million deaths yearly in the globe; also, multidrug-resistant TB and extensively drug-resistant TB in Europe, Asia, and SA have become the most threatening disease globally. WHO commemorated the World TB Day on March 24^th^, 2015, by compiling a specific issue of the International Journal of Infectious Diseases, which incorporates 32 articles with a scope inclusive of tuberculosis-related issues written by a global authorship.

The End Tuberculosis strategy and Global Action Plan on Antimicrobial Resistance were formed to better health and control contagious diseases and thereby mitigate the propagation of AMR. In spite of different organization structure and funding resources, the merger of certain activities could result in better outcomes and achieving mutual goals. In most countries, the End TB strategy acknowledges the significance of collaboration with other inventive programs [123]; this includes collaboration of TB programs with HIV programs [123].

Five tactical aims to attain the objective of securing progress of healthcare management and prevention of contagious sicknesses with effectual and safest medication [124] were developed. The strategies include (1) improving the awareness and understanding of AMR by effective communication, education, and training, (2) enhancing the knowledge and evidence base through surveillance and research, (3) reducing the prevalence infection through effective sanitation, hygiene, and infection prevention measures, (4) reducing the utilization of antimicrobial drugs in human and animal health, and (5) developing the economic case for sustainable investment that takes into account the needs of all the countries and increasing investments in new medicines, diagnostic tools, vaccines, and other interventions [97]. To reduce the strain of resistance, the congress also made some recommendations which included (1) customization of the surveillance and monitoring systems that are used to contain AMR to meet country requirements, (2) regulatory control and risk analysis of human and veterinary medicines, (3) pinpointing the fundamental pathogens and reviewing the intake of antimicrobials, (4) making use of the existing structure to facilitate the coordinating stakeholders such as to participate in tuberculosis and HIV management programs, (5) determining the shortcomings of the surveillance systems in place and rectifying their deficit, and (6) reviewing how the present infection control guidelines add value [11].

All these public health management principles are designed and programmed to attain high success rates during treatment of drug susceptible cases, trying to diagnose and cure the highest possible proportion of cases with MDR-TB while discontinuing any further transmission within the community [125]. The principles put emphasis on the administration of environmental control measures and personal protective equipment such as respirators and masks to prevent healthcare workers from contracting infections from patients and visitors [126–128]. WHO strongly recommends that the admission of MDR-/XDR-TB patients which is not necessary should be reduced in healthcare premises to avail space for patients that are in life-threatening conditions, acute cases, detrimental events, and co-occurring conditions [126–128].

### 3.3. The Impact of the Current Pandemic (COVID-19) on the Trend of AMR and ARGs

The outburst of coronavirus disease 2019 (COVID‐19) is adding more pressure to an existing ARG problem because of the high consumption of antibiotics. Coronavirus disease 2019, a respiratory disease, has been recognized as a major public health concern globally and hence was officially announced as pandemic by the World Health Organization [129]. The coronavirus disease was initially identified in China in 2019 and propagated to more than 150 nations [130]. The virus has shown phylogenetic similarities to SARS‐CoV‐1 which also resulted in SARS epidemic in 2002 [131] and is also called severe acute respiratory syndrome coronavirus.

Currently, there are no therapeutics available, and it is presumed that there is no immunity in the populace. Since there is no therapy that has been proven to be effective, most patients are prescribed antibiotics for treatment and prevention of the supposedly bacterial coinfection. A broad-spectrum macrolide anti-drug azithromycin has been prescribed the most for treating COVID-19 patients in many countries of the world [132, 133] even though there is still no evidence to support its use. WHO released an alert about undiscriminating use of antimicrobial as COVID-19 therapy [134]. This behavior can be easily discontinued in countries with high income because the microbial cultures can be conducted timely. Nevertheless, in Africa, unnecessary antibiotic courses could be carried on for quite some time because of scarce resources. Nonadherence to antimicrobial protocols promotes the selection pressure of AMR [135]. Consequently, the COVID-19 pandemic is anticipated to extend over in countries with heavy burden of HIV and tuberculosis. Therefore, one needs an intensive realization of the interactivity between the three fatal epidemics. WHO has released a statistical review with countries that are facing HIV/TB challenge together with the latest results of the COVID-19 pandemic. The incidents of tuberculosis in sub-Saharan Africa have been predicted to have escalated after the outbreak of COVID-19 for the following countries: SA, Uganda, Nigeria, Zambia, Cameroon, Tanzania, Kenya, and Mozambique [136].

Additionally, since the outbreak of COVID-19, the public has been constantly encouraging high frequency of hand hygiene using soap and water, as well as alcohol-based sanitizers to be used. This is a good hygiene standard practice and reduces the spread of COVID-19 effectively; however, this could result in an increase of AMR. Antimicrobial soaps and disinfectants contain antimicrobials and antibodies, and their high usage will increase their concentration in wastewater and other receiving bodies. Therefore, this might potentially cause a negative impact on our health posing humans to high risk because high concentration of antimicrobials may exacerbate the selection of AMR bacteria [135, 137].

It was also predicted that the COVID‐19 epidemic would probably have an impact on many medicinal product supply networks because on the 27 of February 2020, the U.S. Food and Drug Administration (FDA) released an alert notifying about the shortage of a certain drug due to the COVID-19 pandemic and this was preliminary to the description of the pandemic [138]. On the 21 of March 2020, a list of FDA drugs that are in shortage was issued and the list contained 26 oncology medications [138]. Drug shortage might not manifest immediately unlike other suppliers, but the apprehension of their scarcity would particularly be very difficult to manage [139]. Otherwise, the interruption of drug supply could result in drug resistance due to one of the factors (limited or interrupted drug supply resulting in unintentional monotherapy) that have been discussed in the previous section [140].

Moreover, not much attention has been paid to the fact that the pandemic could have an AMR. Previously, the increased number of *S. aureus* infections and their resistance towards methicillin were observed in medical healthcare centers, and it was associated with high consumption of anti-drug during the pandemic [141, 142]. This therefore highlights that patients that have acquired COVID-19 might have antimicrobial-resistant strains on top of the virus [135].

The COVID-19 epidemic has also impacted TB negatively, as the incident rate increased about 3.6% between the years 2020 and 2021 reverting the 20% progress that was achieved in the past two decades. The pandemic also affected the patients that were under RR-TB and MDR-TB treatment between 2019 and 2020. The supply for the treatment decreased from 181 533 to 150 469 leaving 1 in every 3 patients in need. The supply partially improved by +7.5% in 2021 taking the numbers to 161 746 [6].

### 3.4. Causative Factors of Drug Resistance

#### 3.4.1. The Emergence of Drug Resistance in the Environment

The development of AMR is viewed as one of the main prime health concerns in the 21st century [143] because antibiotics are no longer effective to patients who require them for their intended infectious treatments. The detection of AMR residues in the aquatic environment is hypothesized to cause selection for antimicrobial resistance bacteria (ARB) [115, 144]. For example, it has been reported that anti-tubular drugs are not completely metabolized like any other pharmaceuticals; as a result, they get excreted unaltered in urine or feces into the sewage system [144, 145]. The partially unmetabolized drugs and their residues therefore find their way into wastewater treatment plants (WWTPs) and finally get to surface water bodies which are used in households and agricultural activities. Several studies have suggested that when these anti-tubular drugs get to the waste line, some of their metabolites return to their original formulation in the process of wastewater recycling [146, 147]. Hence, effluents from domestic and medicinal plants are conceived as wellspring of AMR synthesis in coastal environment. Once they reach the aquatic environment, they acquire resistance which they pass on either to animals or human pathogens through the food chain. Pharmacogenomic research has confirmed that about 70% of all antimicrobials ingested are ejected unaffected [115]. The studies elucidate further and estimate that approximately 24% of rifampin is ejected unmetabolized converting to 5.606 kg being discharged to the environment every year in Gauteng province only [148]. The removal efficiency of the antimicrobial compounds that have been discharged to the WWTPs by hospitals, pharmaceutical stores, and households [148] differs according to the kind of process employed for the wastewater recycling and this ranges from 30% to 90% [149, 150]. The physicochemical properties of the antimicrobial compounds also influence their removal efficiency [151]. Most WWTPs even the advanced ones are inefficacious in removing some of the drugs from wastewater [152, 153]; as a result, antimicrobial residues have been identified extensively in wastewater in high volumes. This was associated with intermediate compounds that could have formed when the parent compound is degraded during the treatment process [154]. Regardless of the declarations about the good practices by the manufacturing practices, large quantities of antimicrobial residues have been isolated from wastewater effluents [150]. Consequently, a concentration of 31 mg/L fluoroquinolones has been detected in surface water bodies that receive effluents from the medicinal manufacturing plants in India [155] as well as in the United States of America (USA) [156]. Antivirals and endocrine disrupters were also reported to be present in the oceanic environment in South Africa, and their human health risk was quantified [150, 157–159]. This can be attributed to the nondegradable physiochemical structure of these antimicrobial compounds. Antimicrobial compounds such as isoniazid (INH) and ethambutol (EMB) do not easily degrade and thus are persevered in the aquatic environment [160] and therefore develop antimicrobial resistance in other pathogenic microorganisms [144, 161, 162].

#### 3.4.2. The Emergence of Drug Resistance in Healthcare and Communal Settings

TB antimicrobial resistance can emerge from intra-patient evolution which is also known as acquired resistance or from direct transmission of genetically resistant bacteria which is normally referred to as transmitted resistance [163]. Several *Mycobacterium tuberculosis* outbreaks have been in homeless shelters, prisons, living squatters, and hospitals [164]. All these people end up in healthcare centers. As a result, healthcare centers have become a wellspring of *Mycobacterium tuberculosis* multidrug resistance due to many outbreaks that increase the number of *M. tuberculosis* infections. Notably, the transmission of the *Mycobacterium* often takes place in patients who have not been diagnosed with pulmonary or laryngeal TB and therefore not enrolled in any TB therapies. The increasing number of *M. tuberculosis* infections brings about concerns particularly to people that are HIV/AIDS-positive as they are more likely to contract active *M. tuberculosis* [165]. Regardless of all the efforts made to bring better understanding about the dynamics of *M. tuberculosis* transmission and measures to control its propagation, transmission continues to take place especially in clustered places [164].

## 4. Recommendations, Future Perspectives, Knowledge Gap, and Action Gap

### 4.1. Recommendations and Future Perspectives

Tuberculosis is becoming a worldwide burden by developing resistance to existing drugs and therefore compels for the development of novel anti-TB agents [116]. Instant actions are necessary to help the coverage and improve quality for the methods that will assist for early detection so that effective regimen for drug susceptible TB can be initiated according to WHO treatment guidelines. This is to make certain that patients with antituberculosis resistance are well taken care of and also receive adequate treatment. Also, the fast accumulation of MDR-TB requires the treatment sites that will be able to provide quicker molecular diagnosis strategies as well as the treatments that will mitigate further transmission of the deadliest disease in all countries [78]. Standardized therapies in the presence of the undetected underlying AMR challenge have been determined as the prime driver of the drug resistance [68, 166]. Therefore, the focus should be shifted from other contributing factors such as nonadherence by patients, individual pharmacokinetics [167], and variable penetration of drugs into tuberculous lesions [168, 169] and directed to the prime driver. Another contributing factor to the worldwide burden of drug resistance is the emergence of AMR in wastewater which eventually finds its way back to mammals and human beings through the food chain. Therefore, the surveillance of the pathogens that are present in the effluents that are released into the surface water bodies is very crucial as they create unnecessary challenges to the community especially to children and people with compromised immune system such as people that are HIV-positive [170]. Research about pathogens present in wastewater effluents of SA has been conducted; however, small or no findings about the detection of *Mycobacterium* TB in the treated wastewater effluents are reported. This suggests that South Africa does not have sufficient data concerning resistance profiles of *Mycobacterium* microorganisms isolated from hospital or community sewage; therefore, this area of research requires more attention. Communal lessons should be conducted frequently to substantiate the implementation of compliance and concordance [11]. To improve and limit the dissemination of infection, antimicrobials should not be dispensed without the prescription from an authorized medical practitioner. This will strengthen the legislation and improve the availability of antimicrobials of good quality and also limit illogical use of antibiotics especially in food and animal farming. Surveillance of antibiotics is the key to a national AMR containment program. Risk analysis, contagion transmission management, investigation, intercommunication and indoctrination, surveillance, and closer observation are critical success factors in the management of AMR [11]. It should be understood that antimicrobials are meant to be used when preventive actions of maintaining healthy status and infectious diseases have failed but not as a replacement.

### 4.2. Knowledge Gap

The existence of other pharmaceuticals in oceanic environment in South Africa has been well documented [150, 157, 158, 171]. Nonetheless, the presence of anti-TB drugs in the surface waters has not been thoroughly researched [144]. Therefore, the originality of the antimicrobials in surface waters needs to be inferred so that risks associated with their presence can be reduced [144]. Wastewater treatment plants and farms now serve as an important reservoir of ARGs [172] and there are no scientific justifications of their use as growth enhancers. Thus, more information is required with regard to the use of antimicrobials for such purposes. Tracking and characterization of ARGs in different environments are required to streamline their investigation [173]. Also, humans need to be educated so that they understand that long exposure to antimicrobials can result in the emergence of MDR because the lack of knowledge of antibiotics by the general population as well as healthcare provider adds more pressure in the emergence of ARGs.

### 4.3. Action Gap

**Figure 6:**
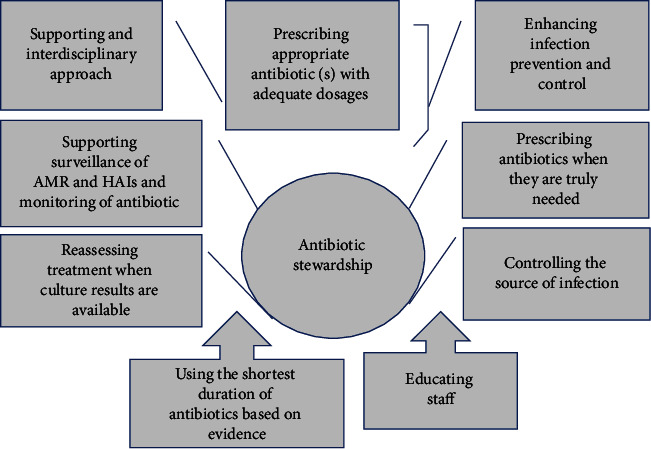
Factors that are controlling the stewardship of antibiotics [135].

**Figure 7:**
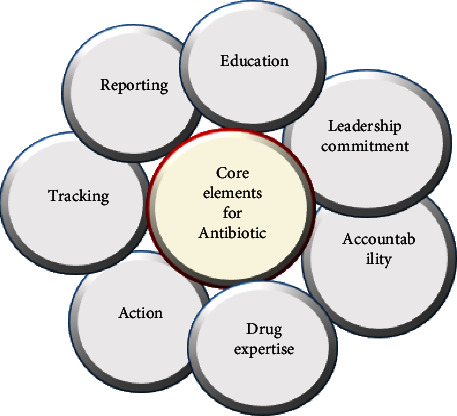
Core elements for antibiotic stewardship [174].

- The stewardship of antibiotics should be enforced to optimize the governmental use of antimicrobial and to reduce the propagation of antimicrobial resistance [135] as presented in Figure 6.
- It is necessary that the government work together with agriculturalists, pertaining NGOs, clientele, vets, physicians, and scientists to address the adversity of using antibiotics inappropriately or unnecessary [11].
- The review to converse about the probable adversities that come along with having the antimicrobials in the environment is necessary.
- The outcome of the review is imperative as it can be used as a determining factor in future studies for waterworks as well as health governance [144].
- Research and development of new antibacterial that will target pathogens which have become resistant to the already existing antimicrobials is required.
- Implementing the core elements that are listed in Figure 7 for antibiotic stewardship is of high importance [174].

### 4.4. Way Forward

- The magnitude and the consequences of the emerged AMR challenge should be quantified in every country including South Africa as it has been identified as a global challenge.
- The surveillance of AMR should be proceeded as it is critical for understanding and controlling the pathogens through relevant health policies; this includes indoctrination, vaccination, and antimicrobial suggestions.
- The surveillance systems need to be designed in a way that will meet the requirements of the country and that includes both economic sectors [11].
- Taking into consideration the COVID-19 epidemic and its compelling physical distancing, strategies for reducing the disruption of TB and HIV therapies should be implemented [129] to ensure that patients continue to receive proper treatment.

## 5. Conclusion

In spite of all the efforts that have been made to combat the curable TB disease, it perseveres to be one of the critical health challenges globally resulting in millions of deaths. The emergence of the AMR exacerbates the situation [175]. The successful outcomes of the TB therapeutic regimens and management are strongly threatened by the evolution and transmission of MDR-TB [35]. The coexistence of HIV with TB stimulates the drug-drug interactions between an anti-TB agent and ARV therapies [116]. Huge successful outcomes have been attained in untangling the mechanisms of AMR; however, a fast and precise diagnosis tool for the detection of AMR is an important requirement to attain the development goals for TB control [35]. It is predicted that MDR-TB emergence is the repercussion of poor treatment regimens [62], as well as the nonadherence to the antibiotic regimens which promotes the selection and growth of resistant microorganisms [78], transmission of AMR strains in congregated environments, poor quality of drugs, inconsistency of drug supply [175–177], indigence, overpopulation, HIV coinfection, diabetes, drinking problems, immunodeficiency, and the consumption of many more drugs [110]. This, therefore, brings challenge to the National Tuberculosis Control Program in several low and middle-income countries (LMIC) compelling strict measures to be employed. Consequently, in the last two decades, WHO and International Union Against Tuberculosis and Lung Disease (IUATLD) designed a project to survey and track the emergence of anti-tubular drug resistance (DR-TB) globally. After the commencement of this project, about 60% of the countries in the globe have implemented the outlines of the campaign [178]. As a result, about 82% of global drug-susceptible TB cases have been determined and treated successfully and the rate of success of the MDR-TB has been documented to be 55% [124, 179]. Nonetheless, antibiotic-resistant TB surveillance still falls short in determining the trends in the weight of antibiotic-resistant TB because there is no clear evidence that indicates whether it is getting better or worse [54, 180–182].

## Figures and Tables

**Table 1:** Mutations resulting in *M*. *tuberculosis* antimicrobial resistance [36, 57].

WHO category	Resistant genes	Anti-TB drug or drug class
First-line agents	*rpoB*	Rifampicin
	*ponA1*	
	*katG*	Rifampicin
		Isoniazid
	*inhA*	Ethionamide
		Isoniazid
	*oxyR-ahpC*	
	*dfrA*	
	*ahpC*	
	*kasA*	
	*furA-katG*	
	*pncA*	Pyrazinamide
	*panD*	
	*rpsA*	
	*embCAB operon*	Ethambutol
	*ubiA*	

Group A	*gyrA/B*	Fluoroquinolones
	*atpE*	Bedaquiline
	*pepQ*	
	*Rev0778*	
	*Rrl*	Linezolid
	*rplC*	

Group B	*Rv0678*	BRD-9327
	*Rv0678*	Clofazimine
	*pepQ*	
	*Rv1979*	
	*Rv2535c*	
	*ndh*	
	*Ald*	Cycloserine, terizidone
	*alr*	
	*ddl*	
	*cycA*	

Group C	*ddn*	Delamanid, pretomanid
	*fgd1*	
	*fbiA/B/C*	
	*crfA*	Imipenem/cilastatin
	*rrs*	Streptomycin
	*rpsl*	
	*rrs*	
	*gidB*	

Group C	*ethA*	Ethionamide, prothionamide
	*ethR*	
	*mshA*	
	*ndh*	
	*inhA*	
	*folC*	Para-aminosalicyclic acid (PAS)
	*dfrA*	
	*thyA/X*	
	*ribD*	

Other medicines	*Eis*	Kanamycin
	*tlyA*	Capreomycin
	*rrl*	Aminoglycosides
	*embB*	Ethambutol

**Table 2:** Antibiotic sensitivity against various pathogenic species [82].

Pathogenic species	Resistance	Antibiotic	Region
*Streptococcus pneumoniae*	>25%	Penicillin	All 6 regions

*Escherichia coli*	>50%	Third-generation cephalosporins	5 out of 6 regions
	100%	Beta-lactam	Africa

Various organisms	Resistant	Vancomycin	All 6 regions
		Third-generation cephalosporins	
		Clindamycin	
		Carbapenems	

## Data Availability

The datasets used and/or analyzed in this study are available from the corresponding author upon reasonable request.

## Supplementary Materials

Supplementary Materials All the relevant data are included either in the paper or Supplementary Materials. Figures 1–7 are also supplemented in the supplementary document.
