# Supplementary material for: Trends and Progress on Antibiotic-Resistant Mycobacterium tuberculosis and Genes in relation to Human Immunodeficiency Virus
Source: Can J Infect Dis Med Microbiol. 2023 Nov 30;2023:6659212. doi: 10.1155/2023/6659212 (PMC10703531; doi:10.1155/2023/6659212)
Supplement: Supplementary Materials — All the relevant data are included either in the paper or Supplementary Materials. Figures 1–7 are also supplemented in the supplementary document. [file 6659212.f1.docx]

Prolonged illness, hospital environment and clinical settings

Lack of supervised treatment

Limited or interrupted drug supply resulting to unintentional monotherapy

Presence of antimicrobial residues in the aquatic environment

Immunosuppressive and other drugs consumption

Inadequate drug prescription by physicians and patient’s nonadherence to suggested treatment regimens

Poor medical management of treatment or inadequate treatment regimens

**Fig. 1** Diagram indicating factors that effectuate the evolution of resistance and thereby result in multidrug resistance [1], [23], [24].

1. Transformation - incorporation of naked DNA

2. Conjugation - bacterial sex

3. Transduction - phage mediation

Extra chromosomal Plasmids

Chromosomal Mutation

Mechanism of Antimicrobial Resistance

MDR promoting factors

- Prolonged illness
- Hospital environment and clinical settings
- Community settings
- Overuse of antibiotics
- Inappropriate choices of antibiotics
- Inadequate dosing
- Poor adherence to treatment guidelines

MDR

XDR

MDR

MDR

Mechanism of Antimicrobial Resistance

Genetic methods

PDR

**Fig. 2** Mechanistic basis of antimicrobial resistance [55], [56].

**Fig. 3** *Mycobacterium* tuberculosis infections national survey in 2008 [66].

**Fig. 4** National tuberculosis drug resistance surveillance between (2001-2002) and (2012-2014) [71], [72].

**Fig. 5** Global annual Tuberculosis infections depicting the surveillance spanning for over a decade [120], [121], [80], [122], [123], [97] [124] [7].

Using the shortest duration of antibiotics based on evidence

Educating staff

Enhancing infection prevention and control

Prescribing appropriate antibiotic(s) with adequate dosage

Enhancing infection prevention and control

Prescribing antibiotics when they are truly needed

Supporting surveillance of AMR and HAIs and monitoring of antibiotics

Reassessing treatment when culture results are available

Antibiotic stewardship

Controlling the source of infection

**Fig. 6** Factors that are controlling the stewardship of antibiotics [139].

Leadership commitment

Accountability

Action

Tracking

Reporting

Education

Drug expertise

Core elements for Antibiotics

**Fig. 7** Core elements for antibiotic stewardship [178].
